# Effectiveness of psychodrama on mental health outcomes based on Chinese samples: A systematic review and meta-analysis of randomized controlled studies

**DOI:** 10.1017/gmh.2024.89

**Published:** 2024-12-02

**Authors:** Xiaohui Wang, Rui Ding, Rui Luo

**Affiliations:** 1 School of Social Work, China University of Labor Relations, Beijing, China; 2 The Chinese University of Hong Kong, Hong Kong, China; 3 Mental Health Education and Counseling Center, China University of Labor Relations, Beijing, China

**Keywords:** psychodrama, randomized controlled trial, intervention research, meta-analysis, mental health, Chinese samples

## Abstract

**Background:**

Psychodrama (PD), supported by extensive global research, is increasingly becoming a vital method for alleviating psychological symptoms and promoting mental well-being in diverse populations across China. However, comprehensive evidence based on rigorous interventions is currently lacking.

**Methods:**

This article systematically reviews the literature on randomized controlled experimental intervention studies of PD in the Chinese Mainland from 1982 to 2023.

**Findings:**

Following the Preferred Reporting Items for Systematic Reviews and Meta-Analyses framework, this article included seven studies (N = 332, 25 effect sizes). The results demonstrate that PD interventions have a promotional effect (standardized mean difference SMD = 0.768, 95% CI [0.591, 0.946]) across different age groups and settings in randomized controlled trial interventions. In accordance with previous literature, we categorized the effect sizes into two major groups: illness reduction (IR) and health promotion (HP). Subgroup analysis based on these two categories revealed consistent findings. In the IR category, the overall effect size was notably significant (SMD = −0.711, 95% CI [−0.976, −0.446]), and in the HP category, the overall effect was also highly significant (SMD = 0.889, 95% CI [0.705, 1.074]). This finding aligns with previous research in other nations, supporting the significant effectiveness of PD as a counseling method in alleviating psychological illnesses and promoting mental health within the Chinese context.

**Conclusion:**

PD serves not only as a therapeutic tool but also as a preventive and developmental intervention. Moving forward, there is a call for increased emphasis on standardized and randomized controlled experimental studies to further the advancement of PD within China.

## Impact statement

Our study constitutes a significant advancement in understanding the effectiveness of psychodrama interventions in addressing mental health challenges within Chinese contexts. Through thorough review and analysis, we have elucidated the substantial impact of psychodrama interventions on mental health outcomes. Our evidence, derived from rigorous randomized controlled trials (RCTs), stands as the most robust aggregation to date, providing compelling support for the efficacy of psychodrama interventions. Furthermore, by categorizing mental health outcomes into illness alleviation and health promotion, our study not only delineates the therapeutic effects of psychodrama but also offers valuable insights for future research endeavors. The systematic synthesis of data through review and meta-analysis underscores the significant benefits of psychodrama in alleviating psychological illnesses and promoting mental health within the Chinese context. This conclusion is particularly crucial given the escalating prevalence of mental health issues in China amidst rapid socioeconomic changes. Our findings highlight the imperative for standardized interventions and stringent research methodologies in China’s mental health landscape. Moreover, our study emphasizes the necessity for culturally adapted and sensitive approaches in delivering psychodrama interventions, considering the distinct sociocultural context of Chinese society. By elucidating the therapeutic potential of psychodrama and advocating for its integration into mental health practice, our research contributes significantly to the global discourse on effective interventions for mental health. Through our comprehensive analysis of RCTs, we appeal to provide evidence-based insights into the mechanisms underlying psychodrama’s efficacy, thereby potentially facilitating strategic planning in mental health care provision. In summary, our study not only demonstrates the effectiveness of psychodrama interventions in China but also highlights the importance of rigorous research methodologies and culturally sensitive approaches in advancing mental health care within diverse cultural contexts.

## Introduction

China’s swift economic growth and urbanization have elevated a substantial part of its populace from poverty yet concurrently introduced novel psychological pressures. According to data from the China Mental Health Survey, the weighted prevalence of any disorder (excluding dementia) stood at 9.3% within the past 12 months and 16.6% over participants’ lifetimes (Huang et al., 2019), establishing China as one of the nations most profoundly affected by mental health-related conditions (Que et al., 2019). Psychodrama (PD), supported by extensive global research, is increasingly becoming vital for alleviating psychological symptoms and promoting mental well-being in diverse populations across China (Sang et al., 2018).

Developed more than a century ago by psychiatrist Moreno, PD in the Western cultural context originated as a form of group therapeutic model known as “deep group psychotherapy” (López-González et al., 2021). Central to the principles of PD is the key concept of spontaneity and creativity (Karp et al., 1998; Tauvon, 2005). Cruz and colleagues identified 11 core techniques: soliloquy, double, mirror, role reversal, resistance interpolation, sculpture, social atom, intermediate objects, games, sociometry and role training (Cruz et al., 2018). It is widely argued that the dramatic recreation of subjective realities in PD empowers clients to role-play different behavioral patterns, thereby leading to therapeutic efficacy (Lim et al., 2021). Since the PD movement in the 1950s, this psychotherapeutic technique has offered a broad range of possibilities when applied to mental healthcare programs, ranging from clinical patients suffering from psychological disorders (Parrish, 1959) to individuals with subclinical symptoms (Jamshidi Nazar et al., 2014) and those seeking developmental promotion (Orkibi et al., 2023). Due to its demonstrated effectiveness in producing therapeutic outcomes through changing role expectations and improving interpersonal skills, PD has received accreditation from the European Association of Psychotherapies and is endorsed as a beneficial health practice by several European governments, including Austria and Hungary (Cruz et al., 2018).

Despite its introduction in the early 1940s, PD’s systematic and ecological development in the Chinese mainland commenced in the 1980s (Sang et al., 2018). Concerns have arisen regarding its adaptability for Chinese clients due to perceived Western cultural roots. Traditional Chinese values, such as social hierarchy and familism, may hinder participants from sharing personal issues comfortably (Hwang, 2006; Sue et al., 2019), and pose challenges to the practice and effectiveness of PD, which advocates change and confrontation (Ji et al., 2010). Thus, adhering to culturally sensitive principles in Chinese PD (Chen and Yo, 2001), practical solutions involve adjusting session dynamics to include a slower warm-up and an extended sharing section (Lai and Tsai, 2014). Moreover, Chinese scholars have innovatively crafted PD models tailored to oriental perspectives and traditional elements. Evidently, Yi Shu PD and campus PD (also known as campus psycho-scene-drama) have gained widespread adoption in China (Deng et al., 2009; Gong, 2012; Tian et al., 2014). The optimization and innovation in a non-Western context significantly contribute to the growing acceptance and practical implementation of PD within the non-Western cultural context, imparting distinct characteristics to the development of PD in China. Existing studies affirm PD’s effectiveness in diverse mental health outcomes, spanning anxiety, depressive disorder, stigma, hope, social avoidance and internet addiction (Sun et al., 2009; Chen and Hu, 2019; Kong, 2019), with applications extending from hospitals (Zhou and Tang, 2002; Liu et al., 2023) to schools (Wang et al., 2021; Ling et al., 2022), communities (Jiang et al., 2005; Wang et al., 2022) and prisons (Guo, 2009).

In summary, PD is emerging as a promising approach to address mental disorders and enhance mental health in the Chinese psychological context. However, despite increasing acceptance, there is a lack of comprehensive research summary and evidence synthesis in this domain. This article aims to consolidate, analyze and disseminate existing evidence regarding the effectiveness of PD on mental health outcomes in Chinese published randomized controlled trials (RCTs) and controlled clinical trials (CCTs). It seeks to both summarize the research on PD in the Chinese context and promote the outlining of potential future directions.

## Reviews

In the hierarchy of evidence, meta-analysis and systematic review play a superior role in assessing the validity of specific research by integrating results from independent studies (Haidich, 2010). An abundance of thorough reviews has collectively accumulated a global evidence base delineating the implications of PD on mental health – both in mitigating mental health issues and fostering mental well-being. Early works, such as Kipper’s in 1978, illuminated the beneficial effects of PD on behavioral retraining and mental health outcomes (Kipper, 1978). Rawlinson’s extensive review in 2000, which remains among one of the largest, unified the affirmative impacts of PD on mental health, touching upon facets like self-esteem and empathy (Rawlinson, 2000). Wieser’s milestone review in 2007, comprising a significant number of 52 studies, manifested substantial results according to the categories defined in the World Health Organization’s ICD-10 criteria (Wieser, 2007). Further research, such as Daemi and Vasegh Rahimparvar’s in 2018, demonstrated that PD effectively enhances mental health among female adolescents (Daemi and Vasegh Rahimparvar, 2018). An inclusive systematic review by Orkibi and Feniger-Schaal in 2019, analyzing 31 studies, indicated that PD research had shown promise in dealing with mental health conditions such as depression and anxiety (Orkibi and Feniger-Schaal, 2019). Recently, Lopez-Gonzalez and colleagues’ 2021 study, encompassing 14 RCTs and a CCT(referred to as a quasi-RCT by the authors), proposed that PD significantly influences a broad range of outcomes, including mitigating symptoms of mental illnesses and promoting subjective well-being and quality of life (López-González et al., 2021). This synthesis of research underscores the potentially considerable influence of PD on mental health interventions.

Despite a wealth of literature summarizing intervention studies, there have been relatively few attempts to systematically evaluate existing evidence across the globe and conduct meta-analyses. So far, three meta-analyses have been published (Kipper and Ritchie, 2003; Wang et al., 2020; Orkibi et al., 2023). However, owing to linguistic constraints, variations in literature quality, differences in research subjects and methodological limitations, a thorough assessment of PD in China reveals a substantial gap in evidence (see Table 1).

**Table 1.** Existing meta-analysis

Study	Included number	Included design	Dependent variable	Independent variable	Register retrieval	Chinese studies
Kipper and Ritchie (2003)	25	RCT	Mental Health	Psychodrama	NR	N
	(N = 1,325)	Non-RCT	Nonpsychological consequence		NR	
Wang et al. (2020)	9	4 RCTs	Depression	Psychodrama	Y	Y
	(N = 620)	3 CCTs 2 unspecified	Anxiety		Y	
Orkibi et al. (2023)	30	18 RCTs	Mental Health Outcomes	Psychodrama	Y	N
					Y	
	(N = 1,567)	12 CCTs		Drama therapy		

*Notes:* NR = not reported.

The first meta-analysis conducted by Kipper, included 25 studies about their dependent measures, with a combined sample of the participants of N = 1,325 from 1965 to 1999 (Kipper and Ritchie, 2003). Based on the Cohen’s *d* effect size calculation, the analysis concluded that PD demonstrates similar or superior improvement compared to commonly reported group psychotherapy. Nevertheless, this literature falls short in providing robust evidence in several respects: 1) Regarding included literature, it does not include studies from China; 2) Concerning research design, it includes designs beyond RCTs (non-RCTs); 3) Procedurally, crucial details about the analysis protocol, including registration status, and key procedures such as complete search strategies, data extraction specifics and bias assessment, are omitted. 4) In terms of results, it includes nonpsychological facets such as conflict resolution and decision-making.

In Wang’s team’s second meta-analysis, nine studies (four RCTs, three CCTs and two unspecified designs, N = 620) were included, revealing that PD has a significant overall effect on reducing depression (standardized mean difference |SMD| = 2.04) and a medium to large overall effect on anxiety, with effect sizes of |SMD| = 1.35 for the SAS and |SMD| = 0.75 for the HAMA in China (Wang et al., 2020). The analysis adhered to the Preferred Reporting Items for Systematic Reviews and Meta-Analyses (PRISMA) framework, registered the review scheme and demonstrated improved research procedures. Additionally, this article marks the first systematic evaluation of the intervention effects of PD in China. However, limitations include: 1) this article focuses solely on depression and anxiety outcomes, neglecting the positive impact on mental health, thus resulting in less comprehensive results; 2) in terms of research design, both RCT and unspecified studies were included, leading to a lack of methodological focus and 3) regarding literature quality, as RCT studies require stringent procedures, the authors did not report the quality of journals included in the analysis, potentially impacting the interpretation of findings.

In the most recent meta-analysis, Orkibi led an international research team, conducted a multilevel meta-analysis on data from 30 studies (18 RCTs, 12 CCTs) comprising 1,567 participants, conducted prior to January 5, 2021 (Orkibi et al., 2023). The results demonstrated an overall medium effect of drama-based therapies with 144 effect sizes considered. Similar to Wang’s study, the research followed the PRISMA framework and displayed rigor in literature retrieval, data extraction and quality assessment. Furthermore, this article primarily included gold-standard RCTs (without stating that random methods are considered CCTS), indicating high-quality literature. However, a limitation emerges: 1) this article simultaneously includes PD and drama therapy, preventing it from serving as isolated evidence for PD and 2) it does not include literature from China, thus lacking evidence from this region.

Generally, the overview of prior systematic reviews and meta-analyses incorporated both RCTs and non-RCT designs, one did not detail methodological procedure (Kipper and Ritchie, 2003); one covered both traditional PD and drama therapy without Chinese samples (Orkibi et al., 2023); one omitted that the mental health effectiveness of PD should encompass two primary aspects: alleviating mental health issues and promoting mental well-being (Wang et al., 2020).

To our knowledge, this study addresses the research gap by being the first to exclusively focus on Chinese PD RCTs and comprehensively review the efficacy of Chinese PD on positive and negative situations. The study aims to achieve the following objectives: (a) synthesizing available evidence on the application of PD in the Chinese mainland, (b) evaluating the overall effects of PD on mental health outcomes in the Chinese mainland and (c) emphasizing the need for standardization in PD interventions and inform strategic planning. Through a thorough analysis of RCTs, this study aims to provide robust evidence regarding the efficacy of PD interventions for improving mental health outcomes in the Chinese mainland.

## Methods

### Protocol and registration

Adhering to the PRISMA guidelines, the protocol for this systematic review and meta-analysis has been registered (PROSPERO ID: CRD42023443638). Moreover, the methodological framework is informed by insights derived from the Cochrane Handbook (Higgins et al., 2019).

### Eligibility criteria

To facilitate a comprehensive evaluation of PD, this study employs the PICOS framework to delineate the eligibility criteria, encompassing five key elements:

1. Participants: The study did not impose demographic restrictions (such as gender or age) on the participants, encompassing individuals suffering from mental disorders and those clinically free of mental disorders.
2. Interventions: The focus was singularly on PD as an independent intervention. The frequency and duration of the PD sessions were not subjected to restrictions. Studies integrating multiple intervention methodologies, such as amalgamating PD with lectures, group counseling or other drama-based therapies, were excluded.
3. Comparisons: The study accommodated a range of control groups, including no-treatment control (commonly the general health education lectures in China) and other interventions. Studies without comparison groups were excluded.
4. Outcomes: The study included all pertinent psychological outcomes, targeting both health promotion (HP) and illness reduction (IR). These outcomes were assessed either through self-reporting or researcher observation, without limitations on the specific scales, questionnaires or other measurement tools utilized for evaluating diverse mental health outcomes. Studies reporting solely behavioral or cognitive outcomes were excluded.
5. Study design: Only RCTs were considered in this study. According to Orkibi et al. (2023), a study failing to clearly depict its randomization process, regardless of the specific randomization methods, was classified as a CCT. Quasi-experimental studies featuring control groups but lacking in randomization or with unspecified grouping methods were excluded.

### Search strategy

The search string encompassed electronic database searches and systematic retrieval. Systematic literature retrieval from Chinese journals and electronic database searches were undertaken, spanning from January 1, 1982 to December 1, 2023. Four dominant Chinese-language databases were accessed, namely the Chinese National Knowledge Infrastructure (CNKI), WanFang Data (WANFANG), China Science and Technology Journal Database (CQVIP) and China Biology Medicine Disc (CBM). To augment the scope of the literature search, three English-language databases were probed for peer-reviewed publications, including Web of Science, PubMed and ScienceDirect.

In this study, consideration was exclusively given to peer-reviewed academic articles published in either Chinese or English. Gray literature, including conference papers, dissertations, public cases and other non-peer-reviewed sources, were purposefully excluded. Methodologies like case studies, teaching techniques and articles employing qualitative research methods, such as interviews, content analysis and one-group pre-test-post-test designs, were also omitted.

Chinese-language publications, to uphold the rigorous standards of RCTs research, followed Runsen Chen’s advice in the Lancet (Qu et al., 2023), and only those disseminated in Chinese core journals were deemed eligible. These specific journals were required to be indexed in one or more of the following databases: the Chinese Science Citation Database (CSCD), the Chinese Social Sciences Citation Index (CSSCI) or the Peking University-Core Journal of China (PKU). Notably, academic literature from Hong Kong, Macao and Taiwan was not included in the search parameters.

In instances where a study reported only time and treatment effects without disclosing post-intervention data for experimental and control groups and attempts to communicate with the author proved futile, the study was excluded from the analysis due to the unavailability of questionnaire data through alternative means.

### Data extraction

The search strategy aimed at titles, abstracts and keyword fields and was augmented by a manual search of recent review literature. Duplicate references were excised from the dataset. Titles, abstracts were independently scrutinized by two authors based on predefined selection criteria, with discrepancies settled via discussion. Two independent reviewers (DR, LR) performed data extraction, employing a predesigned and piloted spreadsheet for this purpose, which adhered to the PICOS framework – detailing participants, interventions, comparisons, outcomes and study designs. Discrepancies were resolved through consensus, and disagreements were resolved by a third independent reviewer (WXH).

### Risk of bias assessment

Recognized as the gold standard for clinical study designs, RCTs may face methodological challenges. To assess bias risk, this study used the Cochrane Collaboration risk-of-bias tool 2.0 (RoB2), covering domains like randomization, intervention deviations, missing data, outcome measurement and result reporting. The methodological quality of the included studies was independently assessed by two reviewers (DR, LR), and discrepancies were resolved by a third reviewer (WXH). Each selected study was evaluated using RoB2, culminating in final results categorized into “low risk,” “some concerns” or “high risk” of bias.

### Assessment of heterogeneity

To ensure meaningful comparisons, this study followed Cochrane guidelines, prioritizing homogeneity in participants, interventions and outcomes for reliable comparisons. Heterogeneity was assessed using Cochran’s Q test and the I^2^ statistic. If the Q statistic had a P-value <0.05 or I^2^ > 50%, it indicated significant statistical heterogeneity among the studies. Conversely, if the P-value was ≥0.05 and I^2^ ≤ 50%, the heterogeneity was considered not significant. High heterogeneity may stem from methodological or outcome assessment differences, suggesting potential bias.

### Publication bias

This study utilized a funnel plot to assess the potential publication bias in the included studies.

### Analyses

**Figure 1.**
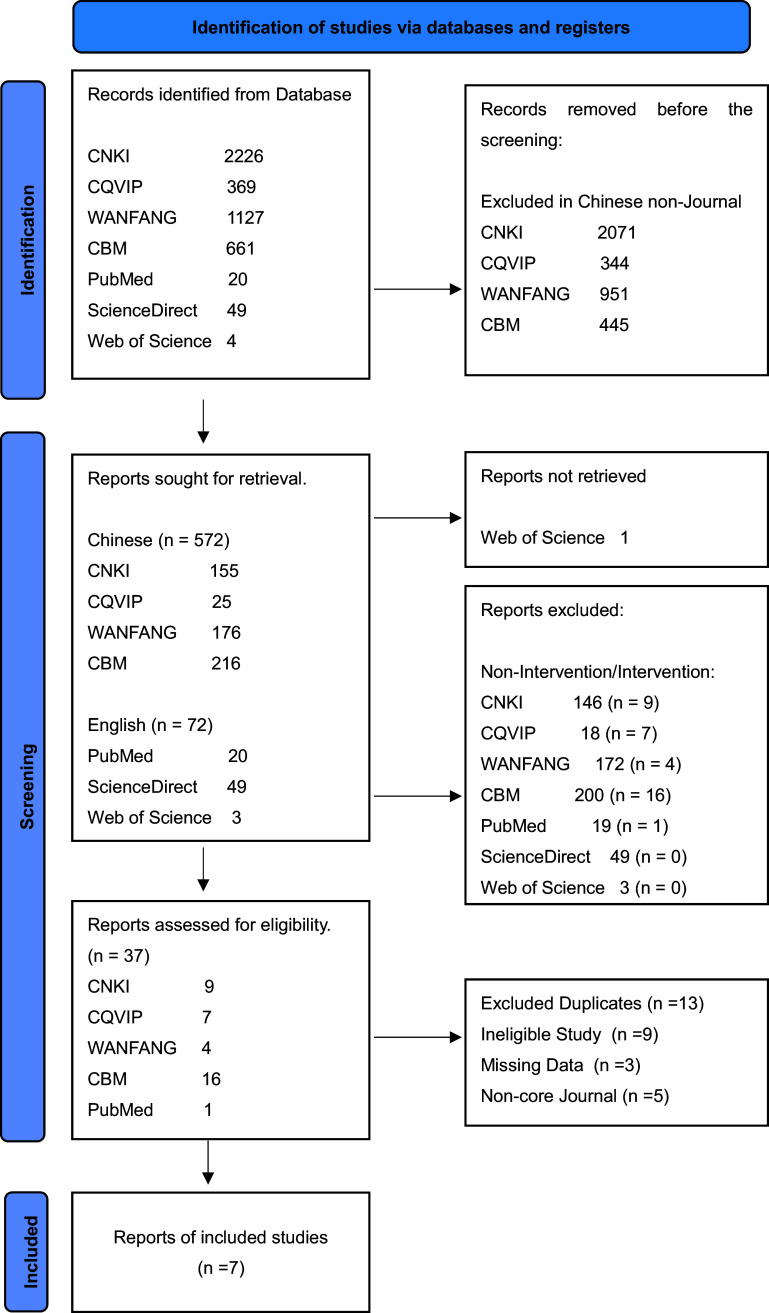
PRISMA flowchart.

Figure 1 illustrates the process of literature selection. A comprehensive search for Chinese RCT studies up to December 1, 2023 identified 4,456 studies across four Chinese databases and three English databases. After eliminating non-journal sources, 644 studies were shortlisted based on titles and abstracts. Subsequent reviews led to the assessment of eligibility for 37 reports after excluding non-intervention articles. Excluded duplicates numbered 13, along with 9 ineligible studies, 3 with missing data and 5 from noncore journals. Ultimately, 7 studies were included, covering 12 psychological health outcomes. Notably, this meta-analysis was deliberately limited to those directly related to mental health. In instances where a single scale encompassed multiple dimensions within a report, only dimensions relevant to mental health were retained.

While some methodologies advocate averaging sample sizes to maintain the overall study count, this approach lacks precision in distributing participants between experimental and control groups, posing potential errors. Additionally, in addressing Study 4 (Wang et al., 2020), only differences as well as baseline means and standard deviations were reported. Endpoint values (means and standard deviations) were estimated using the Cochrane manual formula (Higgins et al., 2019).

Due to significant differences in the rating scales used in the literature, we used the SMD with 95% confidence intervals (CI) as the pooled effect size, with a random-effects model for the meta-analysis. For negative psychological outcomes (IR), an SMD < 0 indicates an improvement in psychological issues following the intervention. For positive psychological outcomes (HP), an SMD > 0 indicates an improvement in psychological issues following the intervention. An absolute SMD value of 0.2–0.5 represents a small effect size, 0.5–0.8 represents a moderate effect size and > 0.8 represents a large effect size (Cohen, 1988). Specifically, meta-analyses were first conducted separately for negative and positive psychological outcomes. These analyses were then combined.

The analysis was performed with Stata 17 software, and statistical significance was determined by a *P*-value <0.05.

For clarity and for the moderation analyses, the type of outcome was grouped into two categories below. Health-promotion outcomes includes Level of hope, Quality of life (only mental health dimension), Mental resilience, General Well-being, Self-esteem, Positive Coping Styles. Illness-reduction includes Depression, Anxiety, Stigma, Internet addiction, Social withdrawal and Negative Coping Styles.

## Results

### Characteristics of PD RCT intervention

#### Target populations

Table 2 outlines the participant characteristics and treatment details from the selected studies; for detailed information, see the Supplementary Material. Among the 332 participants across seven studies, the age distribution varied. One study exclusively focused on the elderly (Cai et al., 2023), four studies on adults (Wang et al., 2020; Yang et al., 2022; Yu et al., 2022; Zheng et al., 2022) and two on children (Du, 2009; Ge et al., 2014). Regarding gender, one study included only male participants (Ge et al., 2014), while four studies included both male and female participants (Wang et al., 2020; Yang et al., 2022; Yu et al., 2022; Cai et al., 2023), with one having gender balance and three showing gender imbalance. One study included only female participants (Zheng et al., 2022), and gender information was not reported in another study (Du, 2009). In terms of health conditions, one study targeted individuals with Parkinson’s disease (Cai et al., 2023); four studies addressed depression (Wang et al., 2020; Yang et al., 2022; Yu et al., 2022; Zheng et al., 2022) (we notice Cai et al., 2023, also measured depression but focused on stigma and hope levels), three of which involved individuals with childhood trauma, and two studies included participants with both psychological and physiological well-being (Du, 2009; Ge et al., 2014).

**Table 2.** Main characteristics of included studies

Authors	Language Index	PD type	Study design	Allocation method	Male Female	N Exp. N Con.	Age group (Exp. and Con.)	Setting	Frequency session	Type of outcome/s
Cai et al. (2023)	Chinese PKU	Classical	CCT	Admission sequence	49/51	50/50	Older adults 71.68 ± 3.47 72.00 ± 3.63	Medical	Sessions of 2 h, every 2 weeks, over 8 weeks.	Depression Anxiety Stigma Level of hope Quality of life
Zheng et al. (2022)	Chinese PKU	Yi Shu	RCT	Table of random numbers	0/47	24/23	Adults 26.42 ± 6.59 26.22 ± 5.98	Medical	Sessions of 4 days, every 2 months, repeated three times over 6 months.	Depression Mental resilience General well-being
Yang et al. (2022)	Chinese PKU CSCD	Yi Shu	RCT	Computer random number	14/54	35/33	Adults 25.49 ± 6.90 27.09 ± 5.68	Medical	Sessions of 4 days, every 2 months, repeated three times over 6 months.	Depression Anxiety Self-esteem Mental resilience
Yu et al. (2022)	English SCI	Yi Shu	RCT	Computer random number	9/37	29/17	Adults 25.970 ± 7.189 28.120 ± 6.214	Medical	Each session lasted for 4 days, once every 2 months, a total of three times for 6 months	Depression Anxiety Coping styles
Wang et al. (2020)	Chinese PKU CSCD	Yi Shu	RCT	Computer random number	4/28	16/16	Adults 28.69 ± 7.552 28.38 ± 6.323	Medical	Each session lasted for 4 days every 2 months, repeated three times over 6 months.	Depression Anxiety Coping styles
Ge et al. (2014)	Chinese PKU CSCD	Classical	RCT	Draw lots	24/0	12/12	Youth 14 ± 0	School	Weekly sessions of 2 h, every Thursday afternoon for 5 weeks.	Internet addiction Social withdrawal
Du (2009)	Chinese PKU	Music	CCT	NR	NR	8/7	Youth NR	Children shelter	Sessions of 60–90 min, four to five times a week, over 5 weeks, totaling 21 sessions.	Self-esteem Anxiety

PD = Psychodrama. NR = Not Reported. RCT = Randomized Controlled Trial. CCT = Controlled Clinical Trial. PKU = Peking University Core Journal Index. CSCD = Chinese Science Citation Database. SCI = Sciences Citation Index.

#### Intervention characteristics

Of the seven studies, four employed Yi Shu PD grounded in the Chinese cultural context (Wang et al., 2020; Yang et al., 2022; Yu et al., 2022; Zheng et al., 2022), one used music-based PD (Du, 2009) and two applied classical PD (Ge et al., 2014; Cai et al., 2023). The interventions varied in frequency, duration and total length, ranging from biweekly 2-h sessions over 8 weeks to sessions of 4 days every 2 months over a period of six months, accommodating diverse schedules and time commitments.

In terms of the phases and methods of implementing PD, four studies adopted the traditional stages of “warm-up, enactment and sharing” (Ge et al., 2014; Yang et al., 2022; Yu et al., 2022; Zheng et al., 2022). One study detailed nine foundational processes of Yi Shu PD (Wang et al., 2020), while two interventions did not report this aspect (Du, 2009; Cai et al., 2023).

Regarding core techniques, five studies reported employing techniques such as role reversal and mirroring (Du, 2009; Ge et al., 2014; Yu et al., 2022; Zheng et al., 2022; Cai et al., 2023), but two did not specify these techniques (Wang et al., 2020; Yang et al., 2022).

Concerning the qualifications of the professionals, five studies detailed the leaders’ credentials. In four of these, the facilitators held relevant certifications (Ge, 2014; Wang et al., 2020; Yang et al., 2022; Yu et al., 2022), whereas one study mentioned a therapeutic team without specifying the qualifications of the therapists (Cai et al., 2023). The remaining two studies did not report on this aspect (Du, 2009; Zheng et al., 2022).

It is noteworthy that four studies employed PD in combination with antidepressant medication as part of a joint treatment approach, with both the observation and experimental groups simultaneously receiving medication (Wang et al., 2020; Yang et al., 2022; Yu et al., 2022; Zheng et al., 2022).

#### Control groups and outcome reporting

In the control groups of the seven studies, five studies provided mental health education (Wang et al., 2020; Yang et al., 2022; Yu et al., 2022; Zheng et al., 2022; Cai et al., 2023), while two utilized a blank control (no care or intervention provided) (Du, 2009; Ge et al., 2014). Additionally, five interventions took place within a medical setting (Wang et al., 2020; Yang et al., 2022; Yu et al., 2022; Zheng et al., 2022; Cai et al., 2023), one in a school-home setting (Ge et al., 2014) and one in a child protection center (Du, 2009).

Across the seven studies, a total of 16 scales were used, including SAS, SDS, SSCI, HHI, PDQ-39, GWB, CTQ-SF, HAMD-17, BAI-21, SES, CD-RISC, BDI-13, TCSQ, CIAS, SAD and FIS. Four interventions, as observed from their intervention descriptions, used self-assessment (Du, 2009; Wang et al., 2020; Yu et al., 2022; Ge et al., 2014), one combined expert evaluation with self-assessment (Yang et al., 2022), one used expert evaluation only (Zheng et al., 2022) and one did not specify the method (based on available details, it likely used self-assessment) (Cai et al., 2023). All studies reported that PD, as an independent variable, had a positive impact on mental health.

#### Experimental design and ethics

Among the seven studies, five were RCTs. Three of these interventions utilized computer allocation for experimental and control groups (Wang et al., 2020; Yang et al., 2022; Yu et al., 2022), one used a drawing lot method (Ge et al., 2014) and one employed a random number table (Zheng et al., 2022). Two studies were classified as CCTs and used the sequence of hospital admissions for assignment (Cai et al., 2023), while one did not specify its design (Du, 2009). Furthermore, only two studies reported follow-up data (at 6 months) (Ge et al., 2014; Yang et al., 2022). In terms of sample size, two studies had a total sample size of up to 50 participants (including both intervention and control group) (Yang et al., 2022; Cai et al., 2023), while the remaining five ranged from 15 to 49 participants (Du, 2009; Ge et al., 2014; Wang et al., 2020; Yu et al., 2022; Zheng et al., 2022). Additionally, four studies reported dropout details (Ge et al., 2014; Yang et al., 2022; Yu et al., 2022; Zheng et al., 2022). Regarding ethics, seven studies obtained informed consent from participants (or from the families of minors). However, three hospital-based interventions reported undergoing an ethical review, with two sharing the same ethical review code (Chongqing Medical University Affiliated First Hospital Ethics Committee Approval, No: 2015290) (Wang et al., 2020; Yang et al., 2022).

### Risk of bias

**Figure 2.**
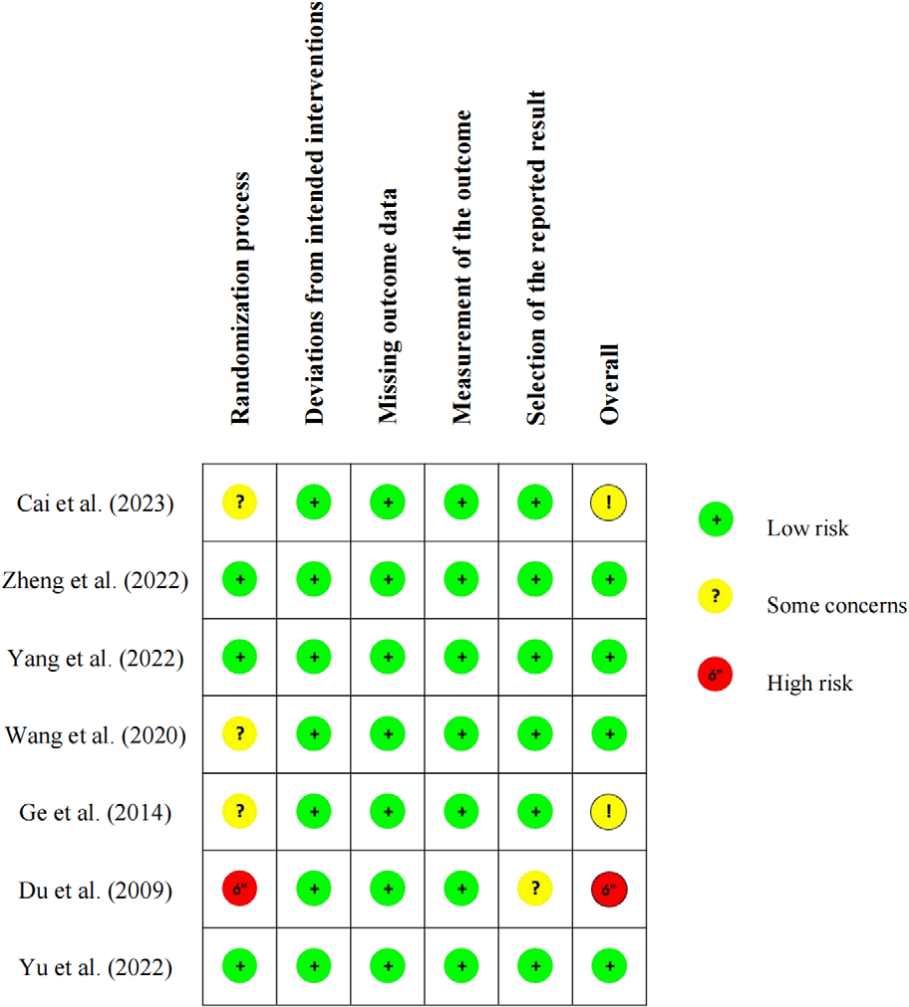
Risk of bias per reviewed study (n = 7).

Figure 2 shows the evaluation results of all included studies. In terms of the randomization process, one study was assessed as high risk because, although the authors emphasized the use of an RCT, the randomization process was not reported (Du, 2009). Three studies had some concerns: one study numbered patients sequentially according to the data (Cai et al., 2023), another had a significantly higher educational background in the intervention group compared to the control group (Wang et al., 2020) and a third used a lottery method which might not have been confidential (Ge et al., 2014). For deviations from intended interventions, missing outcome data and measurement of the outcome, all included studies were considered to be at low risk. In the selection of the reported result, one study (Du, 2009) was considered to raise concerns regarding selective reporting. Overall, the seven included studies were of relatively high quality, as they were all published in Chinese core journals (high-quality peer-reviewed journals). The risk assessment categorized them as high risk (n = 1), low risk (n = 4) and some concerns (n = 2).

### Overall effect of PD on mental health outcomes

**Figure 3.**
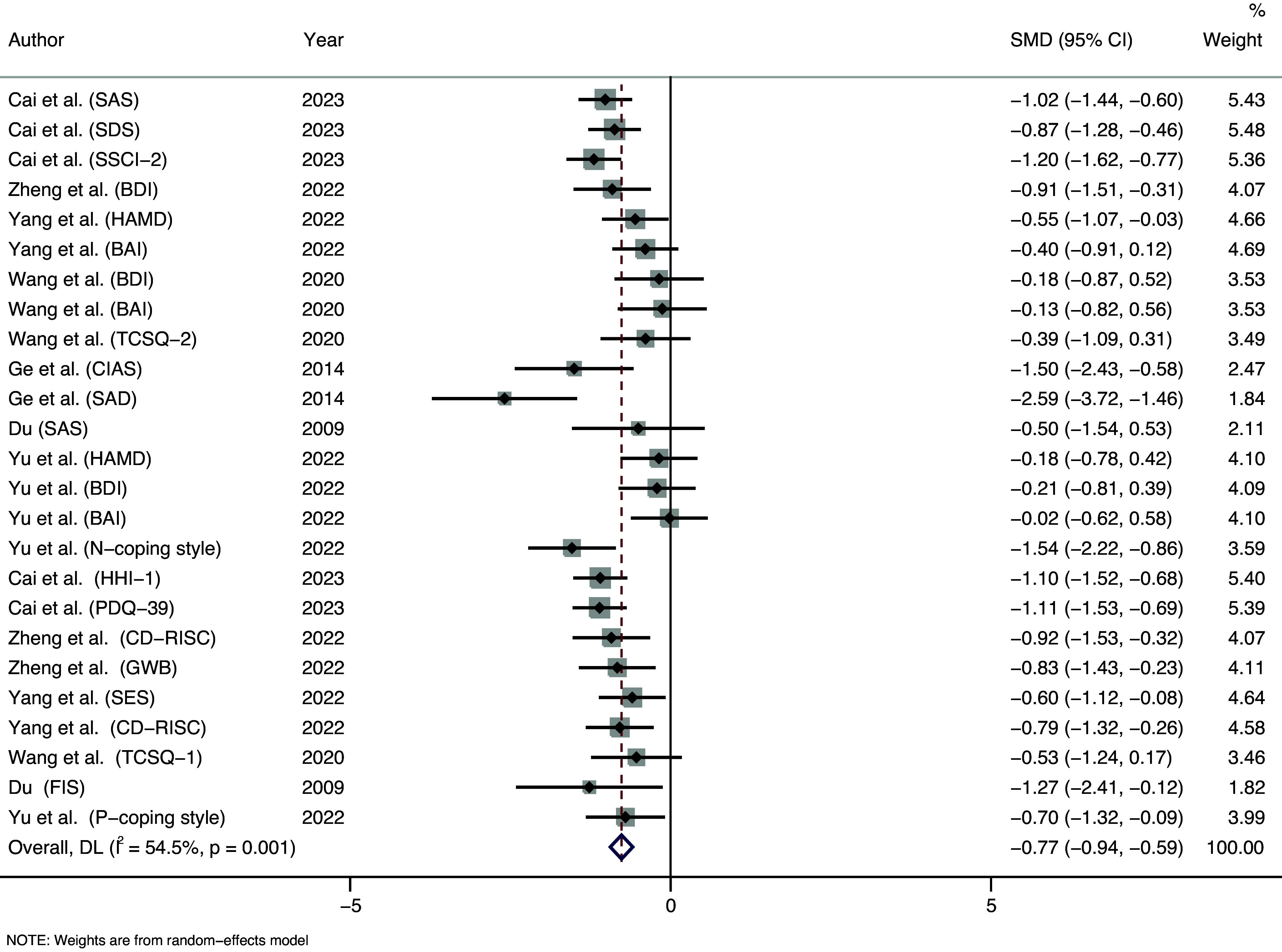
Forest plot of effects on overall mental health outcomes.

The present meta-analysis on the effects of PD therapies on both psychological and behavioral mental health outcomes included 7 independent studies (s), reporting on 25 effect sizes (k), with a total of N = 332 participants. Table 2 presents an overview of the main characteristics of the included studies. Table 3 shows the overall effect of PD therapies on mental health outcomes. Figure 3 depicts a forest plot analyzing the collective efficacy on overall mental health outcomes. Overall, we found a significant medium effect (|SMD| = 0.768, 95% CI [0.591, 0.946]) of PD on mental health outcomes in clinical settings, schools and children’s shelter.

**Table 3.** Overall effect of PD on mental health outcomes

Outcome	S	K	SMD	95% CI	*P*
Illness reduction	7	16	−0.711	[−0.976, −0.446]	*P* < 0.001
Health promotion	6	9	0.889	[0.705, 1.074]	*P* < 0.001
General	7	25	0.768	[0.591, 0.946]	*P* < 0.001

*Note: s = number of studies. k = number of effect sizes. CI = confidence interval.*

*Note: Due to the presence of two directions, the overall effect size was calculated by exchanging the experimental and control groups in the IR calculation.*

### Subgroup analysis

Referring to previous literature, we categorized the effect size into two major groups: IR and HP. Subgroup analysis based on these two categories revealed consistent findings.

**Figure 4.**
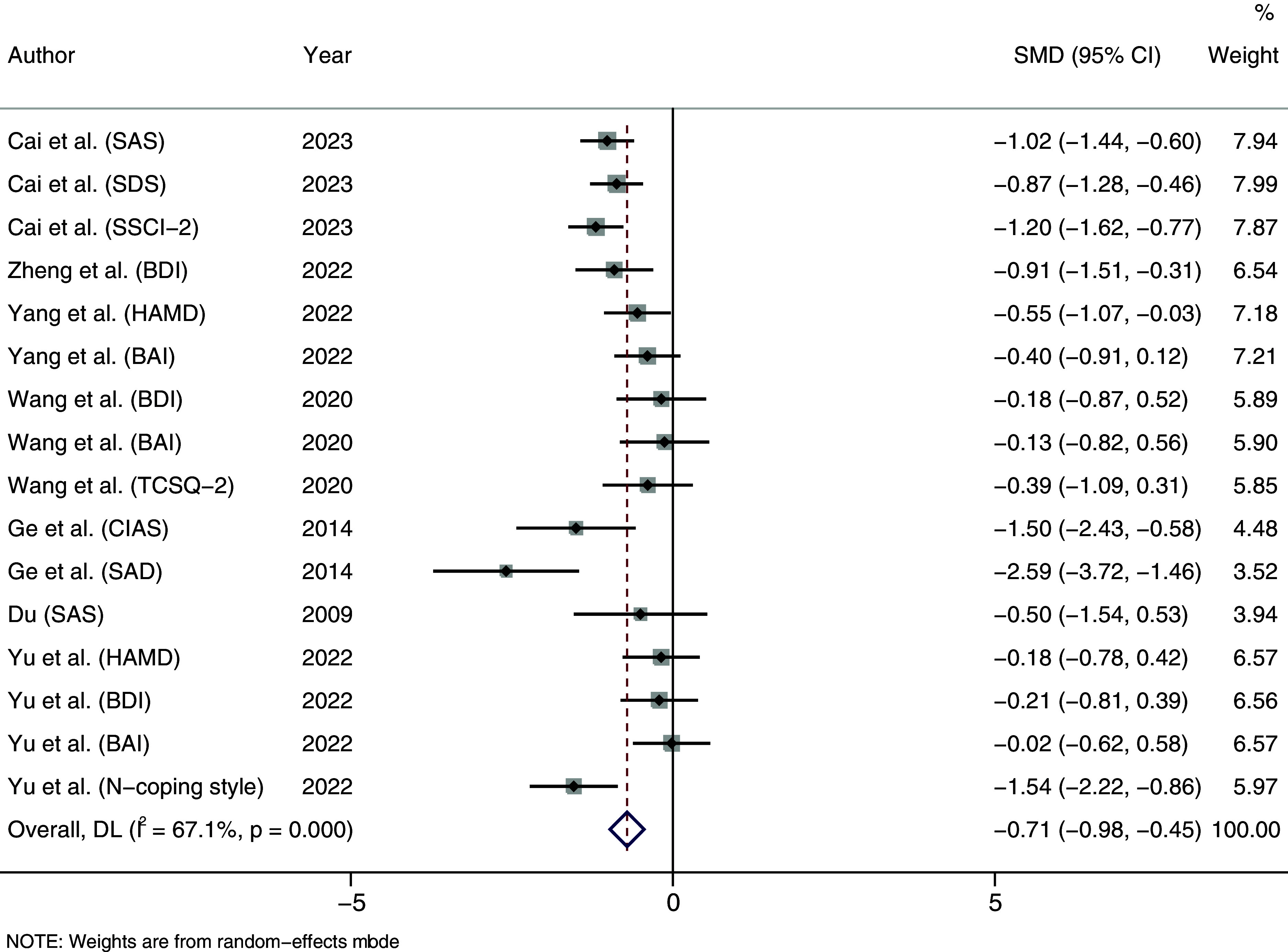
Forest plot of illness reduction.

In the IR category, the overall effect size was notably significant. As shown in Figure 4, the intervention resulted in an SMD (95% CI) of −0.711 ([−0.976, −0.446]), with P < 0.001, indicating a significant decrease in negative psychological scores after the intervention, with a moderate level of improvement.

**Figure 5.**
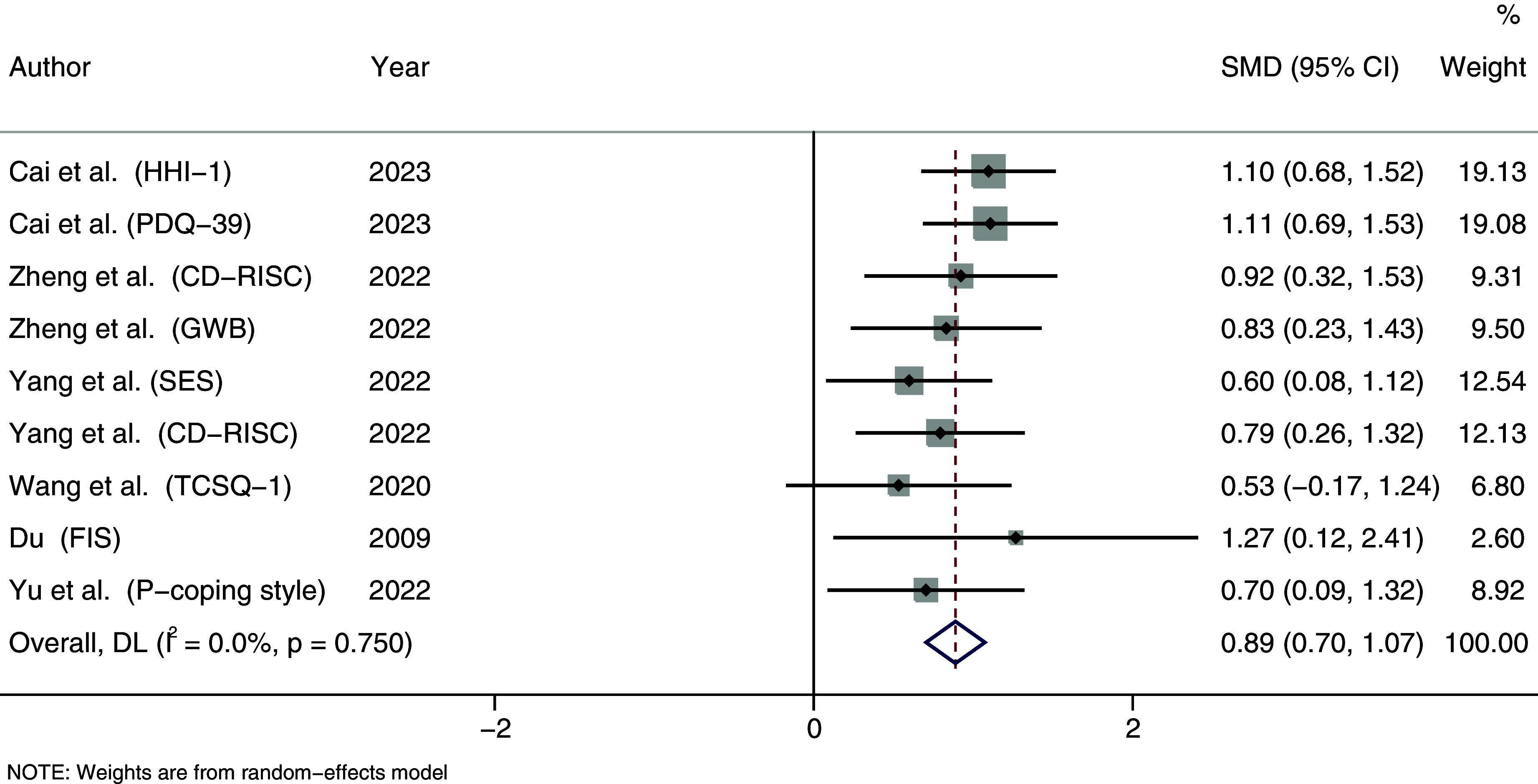
Forest plot of health promotion. Note: The black line represents a confidence interval of the study’s effect size based on the sample size and total number of effect sizes within each study.

In the HP category, the overall effect was also highly significant. As depicted in Figure 5, the intervention led to an SMD (95% CI) of 0.889 ([0.705, 1.074]), with P < 0.001, demonstrating a significant increase in positive psychological scores after the intervention, reflecting a high level of improvement.

This is consistent with previous research, demonstrating that PD, as a counseling approach, has a significant effectiveness in reducing mental illnesses and promoting psychological well-being.

Due to the limited number of eligible studies, conducting Meta-regression or sensitivity analysis for Outcome Characteristics, Study Characteristics, Intervention Characteristics and Sample Characteristics may pose challenges arising from the scarcity of data. Consequently, we refrained from performing further operations such as Meta-regression or sensitivity analysis.

### Bias assessment

**Figure 6.**
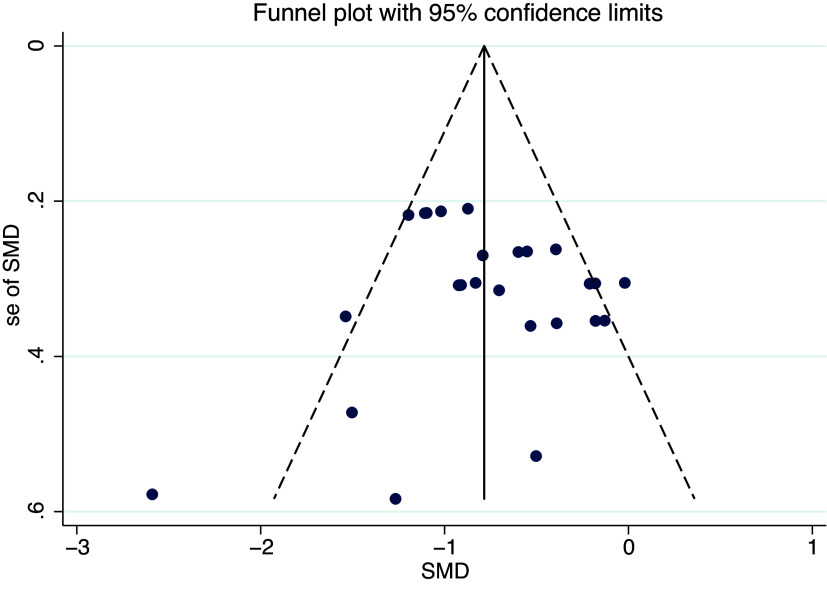
Funnel plot overall.

**Figure 7.**
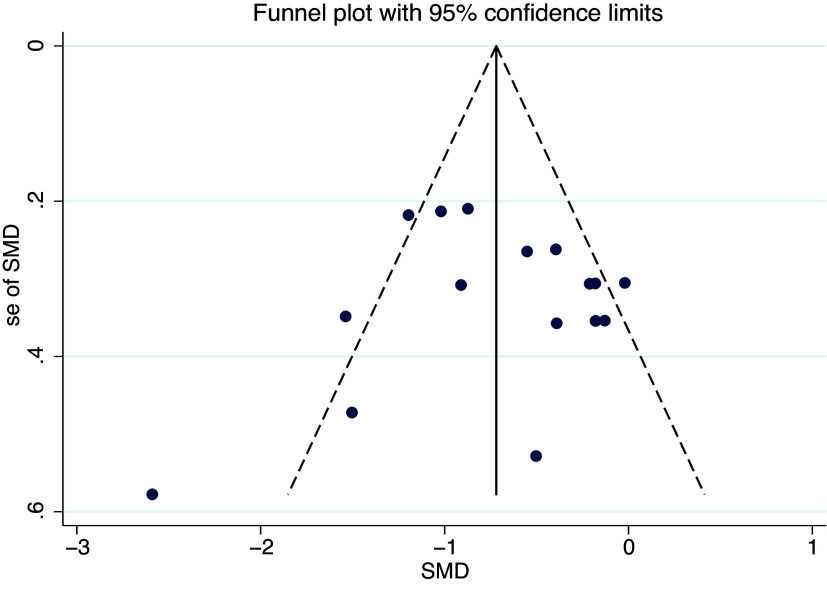
Funnel plot of illness reduction.

**Figure 8.**
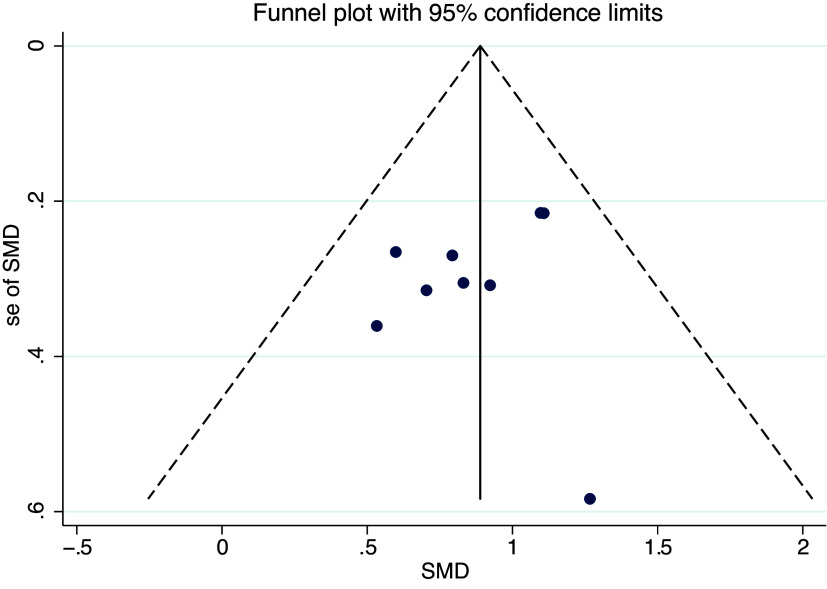
Funnel plot of health promotion.

As shown in Figures 6, 7 and 8, the funnel plot for HP group (Figure 8) shows poor symmetry in the distribution of scatter points, indicating potential publication bias. In contrast, the funnel plots for IR group (Figure 7) and all studies combined (Figure 6) demonstrate good symmetry in scatter point distribution, suggesting no significant publication bias.

## Discussion

Mental health extends far beyond the absence of illness; it is an intrinsic aspect of our individual and collective health and well-being (WHO, 2022). Significant strides have been made in the field of mental health care, leading to the development of evidence-based treatments, including both group and individual therapy approaches. PD is a significant modality among these treatments.

In this study, we systematically reviewed the literature on randomized controlled experimental interventions involving PD in Chinese mainland from 1982 to 2023, excluding studies from Hong Kong, Macao and Taiwan. We methodically categorized, examined and summarized a total of seven RCT/CCT studies, offering insights into the foundational context, intervention strategies and outcomes of PD practices within China. This comprehensive analysis confirms the significant role of PD interventions in both HP and disease reduction. Our findings highlight the positive effects of PD on mental health and well-being, underscoring its value as a therapeutic modality.

### Remaining gap

#### Methodological rigor and sample size

The primary objective of randomized controlled experimental interventions centered on PD is to investigate its influence on selected subjects, evaluating therapeutic efficacy and function. Despite the randomization of intervention groups, several studies display potential concerns in their randomization processes, underscoring an exigent need for more rigorous scientific methodologies in future investigations. The current sample sizes are relatively constrained, ranging from a modest 15 participants to an upper limit of approximately 100, indicating an imperative to augment the sample size in subsequent research endeavors.

#### Technique reporting and integration

PD interventions predominantly adopt a group-based approach, systematically progressing through three stages: warm-up, enactment and sharing. This intricate modality incorporates a diverse range of roles, including a director, protagonist, auxiliary and audience. While some enactments spontaneously unfold, others adhere to preestablished scripts. Contemporary PD exhibits a spectrum of variations, including musical, Yi Shu and campus PD, each underpinned by a primary goal of enhancing participants’ spontaneity and creativity. Core techniques encompass role-play, role reversal, double, mirroring and concretization. These can be seamlessly integrated with artistic mediums such as music, dance or painting and aligned with diverse theoretical frameworks. Notably, a genuine PD intervention should encompass a minimum of three core techniques. To provide a more comprehensive reporting of the genuine therapeutic effects of various PD techniques, greater transparency and discussion regarding the application of techniques in more experiments should be encouraged, with evaluations conducted through both qualitative and quantitative means.

#### Assessment timings and long-term follow-up

A significant majority of studies gauging intervention efficacy via pre- and post-assessments remain vague concerning the specific timings of these evaluations. As a recommendation for future research, we believe that a more detailed delineation of pre- and post-assessment timings is pivotal. Currently, the emphasis predominantly lies on short-term efficacy measurements of PD with a conspicuous dearth of longitudinal follow-ups, underscoring the need for extensive long-term evaluations and periodic follow-up assessments.

#### Standardization in practitioner qualifications

Existing studies varied in their practitioner qualifications: some included professionals with specialized training and directorial qualifications in PD, while others were conducted by experts from diverse fields such as psychiatry, psychological counseling, medicine and education. This lack of standardization in practitioner qualifications highlights a critical gap. Future research should aim to establish consistent standards for PD training and credentialing to ensure the delivery of high-quality practice.

### Contribution

This article is the pioneering study to conduct a systematic review of randomized controlled experimental intervention research on PD in China, providing a reference for future PD intervention research and enhancing practice quality. This article makes a detailed analysis of the basic situation, intervention plan and intervention results of randomized controlled experimental intervention studies on Chinese PD, laying a good foundation for the design and implementation of rigorous intervention studies in the future.

As China’s social psychological service system continues to develop and refine, the potential for applying PD grows significantly. Recognized as a potent method, PD demands practitioners to undergo systematic training to ensure interventions are conducted without causing harm to the individuals involved. To effectively apply PD, practitioners and researchers must collaborate closely, conducting high-quality intervention research to explore its application within the framework of Chinese institutional culture.

### Limitations

In this review, we present affirmative evidence concerning the therapeutic potency of PD. Yet, it is imperative to acknowledge several constraints:

1. Sample size: Even with comprehensive inclusion efforts, a mere seven studies met the criteria for our meta-analysis. According to Hedges and Vevea (1998), when studies number fewer than five, outcomes from random-effects models should be viewed with circumspection. This calls for prudence in effect size interpretations. The paucity of studies further underscores the need for augmented research to refine our comprehension of the overall impact.
2. Study heterogeneity: We noted discernible variation among the included studies, particularly in terms of therapeutic techniques and outcome measurements, with an array of scales employed. The veracity of such analyses is invariably contingent upon the quantity and rigor of the incorporated intervention studies. Therefore, this heterogeneity may potentially limit the interpretative power of our study.
3. Control group inconsistencies: Every study availed itself of a control group. Yet, the divergence in methodology in curating these groups could bolster inter-study variability. This observed variance in control conditions remains a relatively uncharted terrain in the domain of intervention meta-analyses.
4. PD categorization: Several studies showcased the culturally nuanced ‘Yi shu’ PD paradigm, juxtaposing it with its Western counterpart. While Wang et al. (2020) and this study corroborate the salience of this model, its inherent cultural singularity could engender interpretative nuances, especially when offering procedural descriptions to a global readership.
5. Absence of prolonged outcomes: While our foundational objective remained the scrutiny of PD’s interventional impact, it is undeniable that shifts in mental well-being are susceptible to multifarious influences. Thus, follow-up data become quintessential to gauge the genuine and sustained alterations, which was absent in the majority of the included studies.

## Conclusion

Through a systematic literature review, this article summarizes the basic situation, intervention plan and intervention results of randomized controlled experimental intervention studies on PD in China, grasps the research trends in this field and points out future research directions. In general, there are few randomized controlled experimental intervention research results of PD, and high-quality results are also lacking. Future practice needs qualified professional helpers to implement, and more rigorous design, implementation and evaluation are needed in research.

## Supporting information

Wang et al. supplementary material 1 Wang et al. supplementary material

Wang et al. supplementary material 2 Wang et al. supplementary material

## Data Availability

The relevant data extracted for this study are available upon request from the corresponding author. As this study is a systematic review and meta-analysis of published studies, all data are publicly available in the specified references.

## Supplementary material

The supplementary material for this article can be found at http://doi.org/10.1017/gmh.2024.89.
