# Supplementary material for: Effectiveness of psychodrama on mental health outcomes based on Chinese samples: A systematic review and meta-analysis of randomized controlled studies
Source: Glob Ment Health (Camb). 2024 Dec 2;11:e116. doi: 10.1017/gmh.2024.89 (PMC11704573; doi:10.1017/gmh.2024.89)
Supplement: Wang et al. supplementary material 1 — Wang et al. supplementary material [file S205442512400089Xsup001.docx]

**Appendix A. Supplementary data**

Supplementary data related to this article can be found at

Appendix A. Chinese-English bilingual search terms

| Chinese | English |
| --- | --- |
| 心理剧 | Psychodrama, Psycho Drama |
| 戏剧治疗 | Drama Therapy |
| 社会剧 | Sociodrama |
| 表达性艺术治疗 | Expressive Art Therapy |
| 随机 | Random |
| 对照 | Control |
| 实验 | Experiment |
| 随机对照试验 | Randomized Controlled Trial, RCT |
| 临床 | Medical, Clinical |
| 证据 | Evidence |
| 干预，介入 | Intervention |
| 实务，服务，实践 | Practice |

Appendix B. Search strategy

The search was run on 6th December, 2023.

| Databases | Search strategy |
| --- | --- |
| CNKI | (SU %= 心理剧 OR SU %= 戏剧治疗 OR SU %= 社会剧 OR SU %=表达性艺术治疗 OR KY=心理剧 OR KY=戏剧治疗 OR KY=社会剧 OR KY=表达性艺术治疗) AND (SU %= 临床 OR SU %= 证据 OR SU %= 干预 OR SU %= 介入 OR SU %= 实务 OR SU %= 服务 OR SU %= 应用 OR SU %= 实践 OR KY=临床 OR KY=证据 OR KY=干预 OR KY=介入 OR KY=实务 OR KY=服务 OR KY=应用 OR KY=实践) |
| CQVIP | (M=心理剧 OR M=戏剧治疗 OR M=社会剧 OR M=表达性艺术治疗) AND (M=临床 OR M=证据 OR M=干预 OR M=介入 OR M=实务 OR M=服务 OR M=实践 OR M=应用) |
| WanFang | 题名或关键词:(心理剧 or 戏剧治疗 or 社会剧 or 表达性艺术治疗) and 题名或关键词:(临床 or 证据 or 干预 or 介入 or 实务 or 服务 or 实践 or 应用) |
| CBM | (心理剧 OR 戏剧治疗 OR 社会剧 OR 表达性艺术治疗) AND (临床 OR 证据 OR 干预 OR 介入 OR 实务 OR 服务 OR 实践 OR 应用) |
| Web of Science | psychodrama AND china |
| PubMed | psychodrama AND china |
| Science Direct (Elsevier) | psychodrama AND china |
|  | *Only reviews and research articles are selected.* |
|  |  |
