# Supplementary material for: Effectiveness of psychodrama on mental health outcomes based on Chinese samples: A systematic review and meta-analysis of randomized controlled studies
Source: Glob Ment Health (Camb). 2024 Dec 2;11:e116. doi: 10.1017/gmh.2024.89 (PMC11704573; doi:10.1017/gmh.2024.89)
Supplement: Peer review history

# Editor report: Recommendation: Effectiveness of psychodrama on mental health outcomes based on Chinese samples: A systematic review and meta-analysis of randomized controlled studies — R0/PR2

**DOI:** 10.1017/gmh.2024.89.pr2

**Affiliations:** Faculty of Business, Technology, and Education, Ilia State University, Georgia

No accompanying comment.

# Editor report: Decision: Effectiveness of psychodrama on mental health outcomes based on Chinese samples: A systematic review and meta-analysis of randomized controlled studies — R0/PR3

**DOI:** 10.1017/gmh.2024.89.pr3

**Affiliations:** Johns Hopkins University Bloomberg School of Public Health, United States

No accompanying comment.

# Editor report: Recommendation: Effectiveness of psychodrama on mental health outcomes based on Chinese samples: A systematic review and meta-analysis of randomized controlled studies — R1/PR5

**DOI:** 10.1017/gmh.2024.89.pr5

**Affiliations:** Faculty of Business, Technology, and Education, Ilia State University, Georgia

Dear Authors, would you please review Dr. Wieser’s final review/letter and kindly consider his suggestions for the final text of your manuscript?

# Editor report: Decision: Effectiveness of psychodrama on mental health outcomes based on Chinese samples: A systematic review and meta-analysis of randomized controlled studies — R1/PR6

**DOI:** 10.1017/gmh.2024.89.pr6

**Affiliations:** Johns Hopkins University Bloomberg School of Public Health, United States

No accompanying comment.
